# Incidence, Risk Factors and Outcomes of SARS‐CoV‐2 Infection in Pregnant Women: The COROPREG Population‐Based Study

**DOI:** 10.1111/ppe.70028

**Published:** 2025-05-21

**Authors:** Caroline Diguisto, Pierre‐Yves Ancel, Aurélien Seco, Nathalie Baunot, Cecile Caze, Catherine Crenn‐Hébert, Corinne Dupont, Charles Garabedian, Cécile Lebeaux, Camille Le Ray, Mathilde Letouzey, Elsa Lorthe, Emilie Marrer, Valérie Rouger, Christophe Vayssière, Christelle Vauloup Fellous, Marie‐Pierre Bonnet, Catherine Deneux‐Tharaux

**Affiliations:** ^1^ Obstetrical, Perinatal and Pediatric Life Course Epidemiology (OPPALE Team), Centre de Recherche Epidémiologie et StatistiqueS (CRESS), INSERM, INRAE Université Paris Cité and Université Sorbonne Paris Nord Paris France; ^2^ Department of Obstetrics Centre Hospitalier Régional Universitaire et Faculté de Médecine de Tours Tours France; ^3^ Clinical Investigation Center CIC P1419, Assistance Publique‐Hôpitaux de Paris, GH Paris Centre Université Paris Cité Paris France; ^4^ Clinical Research Unit Necker Cochin Assistance Publique‐Hôpitaux de Paris Paris France; ^5^ Réseau de Santé en Périnatalité Parisien Paris France; ^6^ Réseau de Santé en Périnatalité Naître dans l'Est Parisien Paris France; ^7^ Assistance Publique‐Hôpitaux de Paris, Louis Mourier University Hospital Colombes France; ^8^ Research on Healthcare Performance (RESHAPE), INSERM U1290 Université Claude Bernard Lyon 1 Lyon France; ^9^ France Réseau AURORE, Hôpital de la Croix Rousse, Hospices Civils de Lyon Lyon France; ^10^ Department of Obstetrics, ULR 2694‐METRICS CHU Lille, University of Lille Lille France; ^11^ Neonatal Intensive Care Unit Centre Hospitalier Intercommunal de Créteil Créteil France; ^12^ Réseau Perinatal du Val de Marne Créteil France; ^13^ Maternité Port Royal, Hôpital Cochin Port Royal, Assistance Publique‐Hôpitaux de Paris Université Paris Cité Paris France; ^14^ Department of Neonatal Pediatrics, Hôpital Trousseau, Assistance Publique‐Hôpitaux de Paris Sorbonne Université Paris France; ^15^ Unit of Population Epidemiology, Division of Primary Care Medicine Geneva University Hospitals Geneva Switzerland; ^16^ Geneva School of Health Sciences HES‐SO University of Applied Sciences and Arts Western Switzerland Geneva Switzerland; ^17^ Réseau Périnatal Lorrain/Coordination Périnatale Grand Est Nancy France; ^18^ Loire Infant Follow‐Up Team (LIFT) Network Nantes France; ^19^ Department of Obstetrics and Gynecology, Paule de Viguier Hospital Toulouse III University Toulouse France; ^20^ Department of Virology Hopital Paul Brousse Villejuif France; ^21^ Department of Anaesthesiology and Critical Care Medicine, Armand Trousseau Hospital, DMU DREAM, GRC 29, AP‐HP Sorbonne University Paris France

**Keywords:** COVID‐19, maternal morbidity, neonatal morbidity, pregnancy, risk factors, SARS‐CoV‐2

## Abstract

**Background:**

Population‐based data are needed to reliably assess the impact of SARS‐CoV‐2 infection during pregnancy.

**Objectives:**

To estimate the population‐based incidence of SARS‐CoV‐2 infection and its severe forms in the obstetric population, identify risk factors of severe SARS‐CoV‐2 infection (severe COVID‐19) and describe delivery, maternal and neonatal outcomes by disease severity, using a definition of severity based on organ dysfunction.

**Methods:**

A prospective population‐based study conducted over the three first pandemic waves between March 2020 and April 2021 in 281 maternity hospitals in six French regions included all women with SARS‐CoV‐2 infection during pregnancy or within 7 days post‐partum, whether symptomatic or not, hospitalised or not. Severe COVID‐19 forms were defined a priori using clinical, biological and management criteria of organ dysfunction. We calculated infection and severe infection rates and studied associations between sociodemographic, medical and pregnancy characteristics and severe COVID‐19 by univariate and multivariate modified Poisson regression modelling.

**Results:**

From a population of 385,214 deliveries in the participating regions, 6015 women with SARS‐CoV‐2 infection were identified, including 337 severe cases. The rates of severe COVID‐19 were 1.1, 0.9 and 3.6 per 1000 deliveries during the first, second and third pandemic waves, respectively, and the proportions of severe COVID‐19 were 8.6%, 3.4% and 9.3%, respectively. On multivariate analysis, the risk of severe COVID‐19 was associated with younger and older age, migrant status, living with > 4 people, overweight or obesity, chronic hypertension or diabetes and infection ≥ 22 weeks of gestation rather than earlier in pregnancy. Neonatal morbidity occurred mostly with severe maternal infection.

**Conclusion:**

Using an organ‐based definition of severity and population‐based data, rates of severe COVID‐19 appeared lower than in previous studies. A permanent perinatal surveillance system is needed to assess efficiently and rapidly the impact of future pandemics.

## 1 Background

During the COVID‐19 pandemic, SARS‐CoV‐2 infection during pregnancy was rapidly considered a potentially high‐risk situation for the mother and child, especially in view of the existing data for pregnant women during the influenza pandemic [1, 2, 3]. This opinion was reinforced by the first publications on SARS‐CoV‐2 infection in the obstetric population suggesting an increased risk for severe COVID‐19 and even for COVID‐19‐related deaths in pregnant women versus the general population [4, 5]. However, at that time, the quality of the literature was questionable: most studies were not population‐based and were often restricted to hospitalised women, which implied selection bias. Another limitation of the literature was the definition of severe forms of COVID‐19 based on management criteria such as the administration of oxygen or admission to intensive care [6, 7]. These factors likely vary among units and countries, depend on local clinical practices and also evolved rapidly over time according to the rapid changes in care management during this particular period. These limitations bias the estimates of incidence of SARS‐CoV‐2 infection and the frequency of severe COVID‐19 forms and do not allow for firmly identifying subgroups of women at risk of severe forms in the pregnant population.

Although information was urgently needed at the time of the pandemic, we now need to analyse more robust data for an improved understanding of this situation for pregnant women. Indeed, lessons from the pandemic still need to be learned for anticipating future pandemics. Thus, this study aimed to estimate the population‐based rates of severe COVID‐19 in the obstetric population, identify risk factors of severe COVID‐19 among infected women and describe obstetric, maternal and neonatal outcomes according to disease severity, using a definition of severity based on organ dysfunction.

## 2 Methods

### 2.1 Study Design

COROPREG (COROnavirus 2019 infection in PREGnancy) is a French prospective multiregional population‐based study set up at the start of the pandemic to document SARS‐CoV‐2 infection during pregnancy. It included all pregnant women with probable or confirmed SARS‐CoV‐2 infection during pregnancy or within the 7 days post‐partum, whether it led to hospitalisation or not.

**FIGURE 1:**
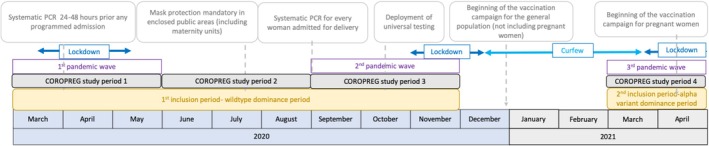
Public health and government measures in France according to the COROPREG study periods.

France experienced three COVID‐19 pandemic waves between March 2020 and April 2021. We defined four study periods: March to May 2020 (first wave, wild‐type variant dominance), June to August 2020 (no wave, low community transmission), September to November 2020 (second wave, wild‐type variant dominance) and March to April 2021 (third wave, alpha variant dominance) (Figure 1). Testing policies changed throughout the study period; initially, testing was mandatory for all women with programmed admissions and became systematic for all women admitted for delivery from September 2020 throughout the whole study (Figure 1). During the first three study periods (March to November 2020), women were included in 281 maternity units of six French regions (Grand‐Est, Hauts‐de‐France, Ile‐de‐France, Occitanie, Pays‐de‐Loire and Rhône‐Alpes‐Auvergne) representing 60% of deliveries in France, with women and unit characteristics close to the national profile [8]. The fourth study period (March to April 2021) included only women in the Ile‐de‐France region and in one area of the Rhône‐Alpes‐Auvergne region because the other regions were missing human resources to continue data collection.

Women with SARS‐CoV‐2 infection were identified by the clinicians of all participating maternity units, either prospectively at the time of their infection when the women were present at the hospital for a prenatal visit or hospital stay, or retrospectively when they reported having met inclusion criteria for a confirmed or probable infection at any time during their pregnancy. Women were informed of the aims and scope of the study. Their oral consent was required to collect data from medical files. We excluded women with legal protection, those who did not receive the information concerning the study, those with a language barrier with whom it was impossible to communicate and those who did not agree to participate. Because ethics authorisation was obtained in May 2020, women who met the inclusion criteria between March and May 2020 were identified by a review of local registers of SARS‐CoV‐2 infection set up in all maternity units; they were then informed by mail and retrospectively included. Research assistants manually reviewed medical and laboratory files of every included woman to complete standardised questionnaires.

### 2.2 Definition of SARS‐CoV‐2 Infection

A confirmed infection was defined as a positive SARS‐CoV‐2 PCR test, and a probable infection was defined as the presence of suggestive clinical signs associated with lung imaging suggestive of SARS‐CoV‐2 infection or clinical signs within 14 days of close contact with a confirmed SARS‐CoV‐2 case as defined by French public health guidelines [9].

Regarding SARS‐CoV‐2 infection characteristics, the trimester of pregnancy was classified as first (≤ 15^+0^ weeks of gestation (WG)), second (15^+0^–28^+6^ WG) or third trimester (≥ 29^+0^ WG). Symptomatic women were defined as those presenting any self‐reported symptom due to their infection. Finally, hospitalisation, intensive care unit admission and death were recorded.

### 2.3 Criteria for Severe COVID‐19 Forms

Severe COVID‐19 forms were defined a priori by a multidisciplinary scientific committee convened by the study coordination team. To overcome the limitations of previously proposed definitions of severe infections based solely on interventions or place of care, the committee developed a composite definition of severe COVID‐19 based on organ clinical, biological and management criteria of organ dysfunction, incorporating criteria proposed by international bodies [10, 11]. Severe COVID‐19 was defined by the presence of any of arterial pH < 7.38, lactate level > 2.0 mmol/L, PaO2 ≤ 80 mmHg, PaO2/FIO2 < 300, continuous positive airway pressure, high‐flow oxygen therapy, invasive mechanical ventilation, clinical diagnosis of acute respiratory distress requiring oxygen therapy, prone position, extracorporeal membrane oxygenation, platelet count < 70,000/mm3, prothrombin time < 60%, plasmatic fibrinogen level < 2 g/L, creatinine level ≥ 110 μmol/L, dialysis and catecholamines administration.

Using the variables ‘severity of infection’ and ‘symptoms’, we divided the COROPREG population into four groups for comparisons: women with a symptomatic‐severe infection, those with a symptomatic non‐severe infection, those who were asymptomatic and those with a non‐severe infection and unknown symptoms.

### 2.4 Perinatal Data Collection

Collected data included maternal characteristics—age, country of birth, health insurance, employment, housing (living with partner, number of people in the household), personal medical history, smoking status and body mass index; pregnancy characteristics—pregnancy achieved by in vitro fertilisation, parity, multiple versus singleton gestation and preeclampsia; delivery characteristics including labour induction, delivery mode, type of anaesthesia, post‐partum haemorrhagic and thrombotic complications; neonate characteristics (gestational age at birth, sex, stillbirth, birthweight and admission to neonatal intensive care unit), after approval of the second parent, if present/declared. Medical characteristics were extracted from medical files; sociodemographic information was gathered by a self‐administered questionnaire completed by the woman.

### 2.5 Statistical Analysis

First, we estimated the rate of SARS‐CoV‐2 infection for each of the four study periods by calculating the ratio of the number of pregnant women with SARS‐CoV‐2 infection to the total number of deliveries over the same period in the maternity hospitals of the participating regions, obtained from the national Hospital Discharge database of the Programme de Médicalisation des Systèmes d'Information (PMSI). The PMSI allows for obtaining the exact numbers of deliveries in units, knowing that less than 0.6% of deliveries occur outside of hospitals in France. The rates of severe COVID‐19 were estimated per 1000 deliveries and per 100 infected women for each of the four study periods. We also calculated and reported 95% confidence intervals (CIs) using the Clopper–Pearson exact method.

Second, the characteristics of women with SARS‐CoV‐2 infection were compared to the 8057 women who gave birth in the participating regions during 1 week in March 2021 without SARS‐CoV‐2 infection during pregnancy (*n* = 510 women excluded), identified from the French 2021 *Enquête Nationale Périnatale* (ENP) database [12]. We applied weighting, taking into account the duration of the COROPREG inclusion period in each region, to accurately represent the source population of the COROPREG survey. Within the COROPREG population, the characteristics of women were described across the four groups by infection severity and symptoms as described above.

Third, we identified the risk factors of severe COVID‐19 in women with SARS‐CoV2 infection (binary outcome variable: severe COVID‐19 group versus all other groups combined) using modified Poisson regression models with a sandwich error term and estimating crude and adjusted risk ratios (RRs) and their 95% CIs. We built three different multivariable regression models to sequentially include potential risk factors: maternal sociodemographic characteristics, maternal pre‐pregnancy medical characteristics and pregnancy characteristics.

Finally, we described delivery, maternal and neonatal outcomes across the four groups of women by infection severity and symptoms.

All statistical analyses involved using Stata 15.1.

### 2.6 Missing Data

There was no missing data on the outcome. The proportion of women with missing data for any variable included in the final multivariable model was 31% and ranged from 0% for study period to 20% for number of people in the household. Details of missing data proportions by variables are reported in Table S2. We assumed that the missing data adhered to a missing‐at‐random mechanism and therefore we imputed them using multiple imputation by chained equations method [13]. We generated 50 independent imputation data sets.

### 2.7 Ethics Statement

The study was approved by the Ethics Committee ‘Comité de Protection des Personnes’ (no. 2020‐A01388‐31 on the 29 May 2020) and the Commission Nationale de l'Informatique et des Libertés (DR‐2020‐222 du 05/06/20).

## 3 Results

From a population of 385,214 deliveries in the participating regions over the study inclusion period, 6222 women met the SARS‐CoV‐2 infection criteria; after excluding 150 women who refused to participate, 54 women who had not been properly informed and 3 women with legal protection, we included 6015 women with SARS‐CoV‐2 infection (Figure S1).

### 3.1 Population‐Based Rates of SARS‐CoV‐2 Infection and Severe COVID‐19 (Figure 2, Table S1)

**FIGURE 2:**
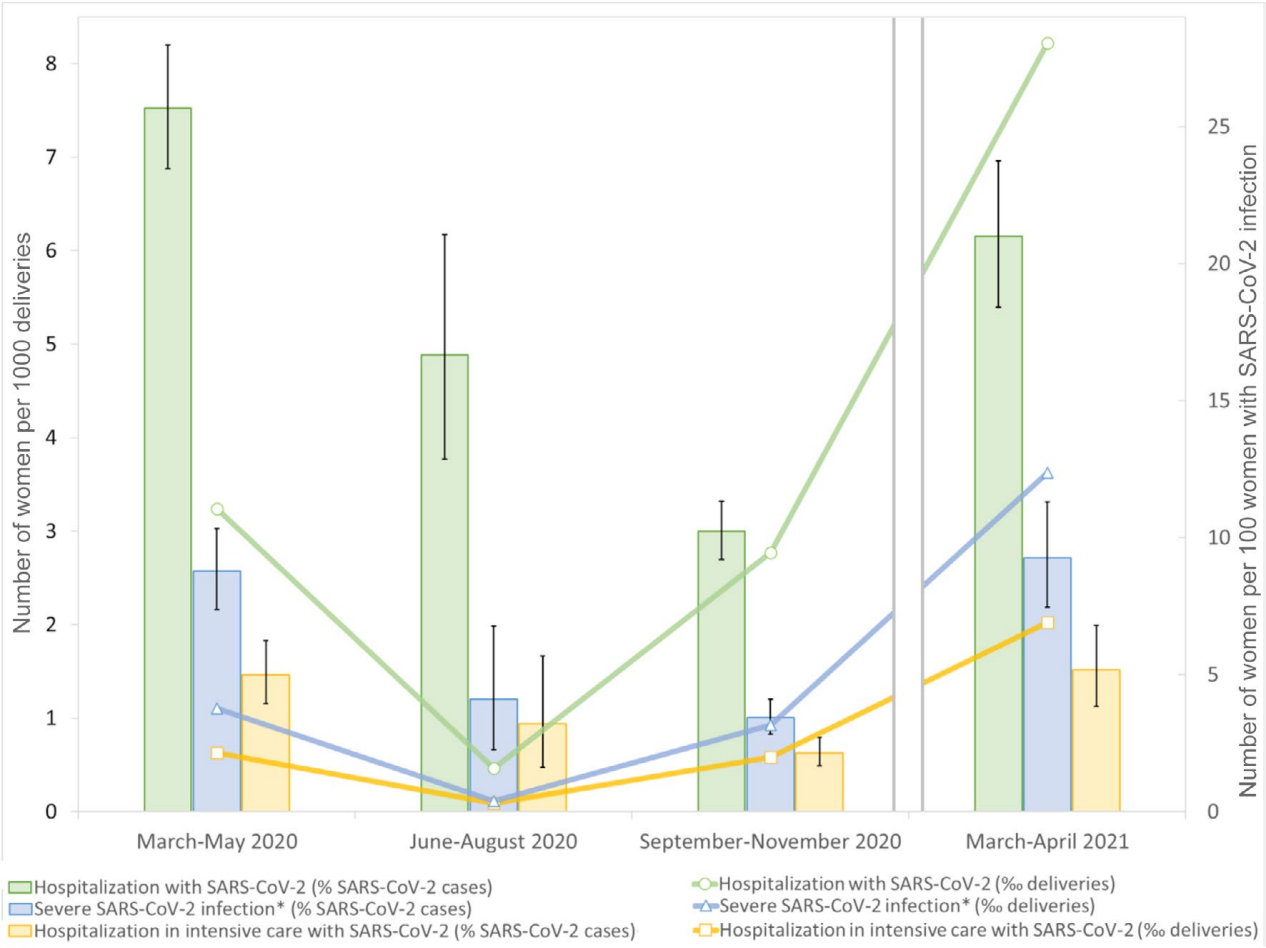
Rates of hospitalisation (green), severe COVID‐19^*^ (blue) and intensive care unit admission (yellow) with obstetric SARS‐CoV‐2 infection, by 1000 deliveries (lines) and by 100 infected women (bars), by study period. ^*^Severe infection was defined by at least one of arterial pH < 7.38, lactate level > 2.0 mmol/L, PaO_2_ ≤ 80 mmHg, PaO_2_/FIO_2_ < 300, continuous positive airway pressure, high‐flow oxygen therapy, invasive mechanical ventilation, clinical diagnosis of acute respiratory distress requiring oxygen therapy, prone position, extracorporeal membrane oxygenation, platelet count < 70,000/mm^3^, prothrombin time < 60%, plasmatic fibrinogen level < 2 g/L, creatinine level ≥ 110 μmol/L, dialysis, catecholamines administration.

The infection rate per 1000 deliveries was 12.6 (95% CI 12.0–13.3) for the first wave (March to May 2020), 2.6 during the low transmission period (June to August 2020) and reached 27.1 (95% CI 26.2–28.0) and 39.2 (95% CI 36.7–41.7) during the second (September to December 2020) and third waves (March to April 2021).

The rates of severe COVID‐19 for the first two pandemic waves with wild‐type variant dominance were 1.1/1000 and 0.9/1000 deliveries and reached 3.6/1000 deliveries for the third pandemic wave with the alpha variant dominance. The proportion of severe COVID‐19 among infected women was 5.6% (337/6015; 95% CI 5.0–6.2) overall, 8.6% (95% CI 7.2–10.1) for the first pandemic wave and 3.4% (95% CI 2.8–4.0) and 9.3% (95% CI 7.5, 11.3) for the second and third waves, respectively.

### 3.2 Characteristics of SARS‐CoV‐2 Infection and Infected Women

The proportion of probable infection was 3% of the study population. While more than half of infections occurred in the last trimester of pregnancy to up to 7 days postpartum, about 1 in 10 occurred in the first trimester, one third in the second trimester, a profile overall stable across inclusion periods (Table 1). The most frequent symptoms were cough, fever and loss or decrease of smell or taste (Table 1).

**TABLE 1:** Characteristics of obstetric SARS‐CoV‐2 infection, overall and by study period.

	*N* ^a^	Overall COROPREG population	March–May 2020	June–August 2020	September–November 2020	March–April 2021
		*N* = 6015	*N* = 1480	*N* = 342	*N* = 3264	*N* = 929
		*n* (%)	*n* (%)	*n* (%)	*n* (%)	*n* (%)
Time of SARS‐CoV‐2 infection diagnosis	6000					
First trimester of pregnancy (until 14^+6^ WG)		775 (12.9)	234 (15.8)	55 (16.1)	410 (12.6)	76 (8.3)
Second trimester of pregnancy (15^+0^–28^+6^ WG)		1829 (30.5)	460 (31.1)	113 (33.0)	987 (30.3)	269 (29.2)
Third trimester of pregnancy (≥ 29^+0^ WG)		3396 (56.6)	783 (53.1)	174 (50.9)	1864 (57.2)	575 (62.5)
29^+0^ to 6 days before delivery		2161 (36.0)	527 (35.7)	126 (36.8)	1206 (37.0)	302 (32.8)
5 days before delivery to 7 days postpartum		1235 (20.6)	256 (17.4)	48 (14.0)	658 (20.2)	273 (29.7)
SARS‐CoV‐2 diagnosis	6015					
Positive SARS‐CoV‐2 PCR test		5843 (97.1)	1346 (90.9)	339 (99.1)	3241 (99.3)	917 (98.7)
Suggestive clinical signs and lung imaging		57 (0.9)	49 (3.3)	1 (0.3)	5 (0.2)	2 (0.2)
Clinical signs within 14 days of close contact with a confirmed SARS‐CoV‐2 case		115 (1.9)	85 (5.7)	2 (0.6)	18 (0.6)	10 (1.1)
Severity of infection^b^	6015					
Asymptomatic		1126 (18.7)	106 (7.2)	80 (23.4)	710 (21.8)	230 (24.8)
Non‐severe infection, symptoms not known		681 (11.3)	140 (9.5)	57 (16.7)	355 (10.9)	129 (13.9)
Non‐severe symptomatic infection		3871 (64.4)	1107 (74.8)	191 (55.8)	2089 (64.0)	484 (52.1)
Severe infection		337 (5.6)	127 (8.6)	14 (4.1)	110 (3.4)	86 (9.3)
Symptoms among symptomatic infections^c^	4208					
Cough	4105	2044 (49.8)	763 (62.8)	89 (44.1)	872 (40.9)	320 (57.6)
Fever or fever feeling	4112	1928 (46.9)	643 (53.0)	105 (51.7)	887 (41.5)	293 (52.6)
Loss or decrease of smell or taste	4098	1917 (46.8)	502 (41.5)	94 (46.8)	1171 (54.9)	150 (27.1)
Severe fatigue or lethargy	4082	1604 (39.3)	387 (32.3)	98 (48.5)	889 (41.8)	230 (41.4)
Headache	4050	1251 (30.9)	301 (25.2)	65 (32.2)	698 (33.1)	187 (34.2)
Rhinorrhea	4056	1077 (26.6)	282 (23.6)	43 (21.5)	644 (30.5)	108 (19.6)
Joint or muscular pain	4063	1078 (26.5)	352 (29.4)	62 (30.8)	488 (23.1)	176 (31.9)
Dyspnea	4101	1056 (25.7)	400 (33.0)	53 (26.2)	406 (19.1)	197 (35.4)
Sore throat	4037	487 (12.1)	140 (11.7)	27 (13.4)	260 (12.4)	60 (11.0)
Nausea or vomiting	4077	397 (9.7)	127 (10.5)	15 (7.5)	173 (8.2)	82 (14.9)
Diarrhoea	4074	326 (8.0)	129 (10.7)	23 (11.4)	145 (6.9)	29 (5.3)
Chest pain	4074	259 (6.4)	85 (7.1)	15 (7.4)	108 (5.1)	51 (9.2)
Other	4114	161 (3.9)	44 (3.6)	14 (6.9)	75 (3.5)	28 (5.0)
Maternal hospitalisation with SARS‐CoV‐2 infection	5987	966 (16.1)	380 (25.8)	57 (16.8)	334 (10.3)	195 (21.1)
Maternal ICU hospitalisation with SARS‐CoV‐2 infection	5989	203 (3.4)	74 (5.0)	11 (3.2)	70 (2.2)	48 (5.2)
Maternal death^d^	6015	4 (0.1)	2 (0.1)	0 (0.0)	2 (0.1)	0 (0.0)

Abbreviations: ICU, intensive care unit; WG, weeks of gestation.

^a^ Total number of available observations for each variable.

^b^ Severe infection was defined by at least one of arterial pH < 7.38, lactate level > 2.0 mmol/L, PaO2 ≤ 80 mmHg, PaO2/FIO2 < 300, continuous positive airway pressure, high‐flow oxygen therapy, invasive mechanical ventilation, clinical diagnosis of acute respiratory distress requiring oxygen therapy, prone position, extracorporeal membrane oxygenation, platelet count < 70,000/mm3, prothrombin time < 60%, plasmatic fibrinogen level < 2 g/l, creatinine level ≥ 110 μmol/L, dialysis, catecholamines administration.

^c^ Each woman could present several symptoms.

^d^ Three maternal deaths were due to severe COVID‐19 with acute respiratory distress and another maternal death not related to SARS‐CoV‐2.

As compared with women from the ENP reference population, women with SARS‐CoV‐2 infection more often were born in North Africa or in Sub‐Saharan Africa, did not have standard health insurance, were more frequently overweight and obese and more often had chronic hypertension and chronic diabetes (Table 2).

**TABLE 2:** Maternal characteristics according to the symptoms and severity of SARS‐CoV‐2 infection.

	*N* ^a^	Comparison of four groups of women within the COROPREG population by symptoms and severity				Comparison of the COROPREG population to reference population	
		Severe infection	Non‐severe symptomatic infection	Non‐severe infection, symptoms not known	Asymptomatic	Overall COROPREG population	Reference population^c^
		*N* = 337	*N* = 3871	*N* = 681	*N* = 1126	*N* = 6015	*N* = 8057
		*n* (%)	*n* (%)	*n* (%)	*n* (%)	*n* (%)	%
Socio‐demographic characteristics							
Age (years)	6012						
<20		6 (1.8)	44 (1.1)	9 (1.3)	26 (2.3)	85 (1.4)	1.1
20–24		40 (11.9)	422 (10.9)	72 (10.6)	141 (12.5)	675 (11.2)	10.0
25–29		57 (16.9)	1179 (30.5)	209 (30.8)	340 (30.2)	1785 (29.7)	27.5
30–34		123 (36.5)	1365 (35.3)	239 (35.2)	384 (34.1)	2111 (35.1)	36.1
35–39		83 (24.6)	686 (17.7)	125 (18.4)	185 (16.4)	1079 (17.9)	19.4
≥ 40		28 (8.3)	175 (4.5)	25 (3.7)	49 (4.4)	277 (4.6)	5.9
Region or country of birth	5705						
France		153 (47.7)	2530 (68.3)	458 (72.7)	671 (63.8)	3812 (66.8)	76.0
Other European country		20 (6.2)	153 (4.1)	26 (4.1)	53 (5.0)	252 (4.4)	3.9
North Africa		52 (16.2)	454 (12.3)	77 (12.2)	132 (12.5)	715 (12.5)	8.3
Sub‐Saharan Africa		70 (21.8)	389 (10.5)	50 (7.9)	138 (13.1)	647 (11.3)	7.4
Other		26 (8.1)	176 (4.8)	19 (3.0)	58 (5.5)	279 (4.9)	4.3
Profession in contact with the public	5208	97 (35.8)	1445 (42.0)	232 (41.8)	399 (42.4)	2173 (41.7)	NA
No standard healthcare insurance	5555	61 (20.1)	361 (10.1)	51 (8.4)	151 (14.3)	624 (11.2)	3.1
Living without a partner	5416	22 (7.2)	173 (4.9)	17 (3.0)	74 (7.2)	286 (5.3)	5.1
Unemployment at the beginning of pregnancy	5307	41 (14.2)	313 (9.0)	61 (10.8)	89 (9.3)	504 (9.5)	12.6
No personal housing	5169	31 (10.7)	225 (6.8)	18 (3.4)	92 (9.1)	368 (7.1)	7.2
More than 4 people living in the household^b^	4812	58 (21.8)	314 (10.1)	58 (11.3)	89 (9.7)	519 (10.8)	10.3
Smoking during pregnancy	5618	16 (5.2)	281 (7.7)	55 (9.3)	98 (9.4)	450 (8.0)	19.1
Medical history							
BMI (kg/m^2^)	5747						
< 18.5		4 (1.3)	140 (3.8)	20 (3.2)	50 (4.6)	214 (3.7)	5.7
18.5–24.9		86 (27.6)	1859 (49.9)	318 (51.0)	546 (50.5)	2809 (48.9)	56.7
25–29.9		96 (30.8)	989 (26.5)	172 (27.6)	283 (26.2)	1540 (26.8)	23.3
30–34.9		84 (26.9)	490 (13.1)	82 (13.1)	136 (12.6)	792 (13.8)	9.3
35–39.9		26 (8.3)	178 (4.8)	25 (4.0)	51 (4.7)	280 (4.9)	3.6
≥ 40		16 (5.1)	73 (2.0)	7 (1.1)	16 (1.5)	112 (1.9)	1.4
Chronic hypertension	5954	17 (5.1)	48 (1.2)	4 (0.6)	17 (1.5)	86 (1.4)	1.0
Diabetes	5954	17 (5.1)	41 (1.1)	7 (1.1)	16 (1.4)	81 (1.4)	0.6
Type I	5954	7 (2.1)	15 (0.4)	5 (0.8)	6 (0.5)	33 (0.6)	0.3
Type II	5954	10 (3.0)	26 (0.7)	2 (0.3)	10 (0.9)	48 (0.8)	0.3
Asthma requiring regular inhaled or oral corticosteroids	5951	17 (5.1)	134 (3.5)	9 (1.4)	22 (2.0)	182 (3.1)	NA
Chronic heart disease	5951	4 (1.2)	51 (1.3)	6 (0.9)	14 (1.3)	75 (1.3)	NA
Autoimmune disease	5951	7 (2.1)	69 (1.8)	15 (2.3)	20 (1.8)	111 (1.9)	NA
Chronic inflammatory disease	5951	5 (1.5)	64 (1.7)	11 (1.7)	10 (0.9)	90 (1.5)	NA
Acquired or induced immunodepression	5955	3 (0.9)	24 (0.6)	4 (0.6)	6 (0.5)	37 (0.6)	NA
Pregnancy characteristics							
Parity	5971						
0		82 (24.4)	1500 (38.9)	242 (37.0)	447 (39.8)	2271 (38.0)	41.3
1		93 (27.7)	1252 (32.4)	217 (33.2)	367 (32.7)	1929 (32.3)	34.4
2		72 (21.4)	676 (17.5)	112 (17.1)	178 (15.9)	1038 (17.4)	14.8
3		48 (14.3)	273 (7.1)	56 (8.6)	80 (7.1)	457 (7.7)	5.9
≥ 4		41 (12.2)	158 (4.1)	27 (4.1)	50 (4.5)	276 (4.6)	3.6
Multiple pregnancy	6001	10 (3.0)	75 (1.9)	8 (1.2)	13 (1.2)	106 (1.8)	1.6
In vitro fertilisation	5840	8 (2.5)	115 (3.0)	14 (2.1)	34 (3.1)	171 (2.9)	3.1

Abbreviations: BMI, body mass index; NA, not available.

^a^ Total number of available observations for each variable.

^b^ 4 is the 75th percentile of the distribution of the number of people living in the household in the overall COROPREG population.

^c^ Women without SARS‐CoV‐2 infection from the *Enquête Nationale Périnatale* (ENP) 2021 population restricted to COROPREG regions and with weights assigned in order to accurately represent the source population of the COROPREG survey.

### 3.3 Risk Factors of Severe COVID‐19 Among Infected Women

On multivariable analysis, the risk of severe COVID‐19 among infected women varied with maternal age with a U‐shape association; was increased in women born in European countries other than France, Sub‐Saharan Africa, or in other countries as compared with those born in France; and in women living with > 4 people in the household (Table 3). After adjustment for other sociodemographic factors, the risk of severe COVID‐19 was positively associated with BMI, chronic hypertension and type I or II diabetes. Women whose infection occurred between 22^+0^ and 28^+6^ WG and from 29^+0^ and later in pregnancy were at increased risk of severe COVID‐19 when compared to women infected in their first trimester.

**TABLE 3:** Associations between sociodemographic, medical, pregnancy and infection characteristics and severity of SARS‐CoV‐2 infection.

	Severe infection	Univariable	Adjusted for sociodemographic characteristics	Adjusted for sociodemographic, medical and pregnancy characteristics	Adjusted for sociodemographic, medical, pregnancy and infection characteristics
	*N* (row %^a^)	RR (95% CI)	aRR (95% CI)	aRR (95% CI)	aRR (95% CI)
	337 (5.6)	*N* = 6015	*N* = 6015	*N* = 6015	*N* = 6015
Sociodemographic characteristics					
Maternal age (years)					
< 20	6 (7.1)	2.21 (0.98–4.98)	1.90 (0.85–4.24)	2.27 (1.04–4.96)	2.04 (0.94–4.43)
20–24	40 (5.9)	1.86 (1.25–2.75)	1.77 (1.20–2.63)	1.78 (1.20–2.64)	1.60 (1.08–2.38)
25–29	57 (3.2)	1.00	1.00	1.00	1.00
30–34	123 (5.8)	1.82 (1.34–2.48)	1.80 (1.32–2.45)	1.74 (1.28–2.36)	1.70 (1.25–2.30)
35–39	83 (7.7)	2.41 (1.73–3.35)	2.13 (1.52–2.97)	1.95 (1.40–2.72)	1.81 (1.30–2.52)
≥ 40	28 (10.1)	3.17 (2.05–4.89)	2.56 (1.65–3.98)	2.22 (1.43–3.43)	1.98 (1.28–3.07)
Region or country of birth					
France	153 (4.0)	1.00	1.00	1.00	1.00
Other European country	20 (7.9)	2.00 (1.28–3.12)	1.76 (1.13–2.75)	1.85 (1.20–2.87)	1.64 (1.06–2.65)
North Africa	52 (7.3)	1.80 (1.33–2.45)	1.51 (1.10–2.09)	1.34 (0.97–1.86)	1.22 (0.88–1.69)
Sub‐Saharan Africa	70 (10.8)	2.67 (2.04–3.49)	2.14 (1.55–2.94)	1.81 (1.31–2.49)	1.40 (1.01–1.94)
Other	26 (9.3)	2.32 (1.56–3.46)	1.94 (1.28–2.94)	1.89 (1.24–2.87)	1.69 (1.13–2.52)
Living without a partner					
No	282 (5.5)	1.00	1.00	1.00	1.00
Yes	22 (7.7)	1.42 (0.94–2.16)	1.13 (0.71–1.80)	1.10 (0.70–1.73)	1.06 (0.67–1.67)
Standard healthcare insurance					
Yes	242 (4.9)	1.00	1.00	1.00	1.00
No	61 (9.8)	1.95 (1.49–2.54)	1.37 (0.99–1.89)	1.26 (0.91–1.73)	1.24 (0.90–1.71)
Personal housing					
Yes	259 (5.4)	1.00	1.00	1.00	1.00
No	31 (8.4)	1.56 (1.10–2.21)	0.91 (0.59–1.41)	0.90 (0.59–1.38)	0.84 (0.55–1.29)
More than 4 people living in the household					
No	208 (4.8)	1.00	1.00	1.00	1.00
Yes	58 (11.2)	2.26 (1.71–2.98)	1.63 (1.20–2.21)	1.35 (0.99–1.83)	1.35 (1.00–1.83)
Medical and pregnancy characteristics					
BMI (kg/m^2^)					
< 18.5	4 (1.9)	0.61 (0.22–1.65)		0.64 (0.24–1.74)	0.62 (0.23–1.68)
18.5–24.9	86 (3.1)	1.00		1.00	1.00
25–29.9	96 (6.2)	2.04 (1.54–2.71)		1.77 (1.32–2.37)	1.71 (1.27–2.29)
30–34.9	84 (10.6)	3.45 (2.59–4.60)		2.78 (2.06–3.77)	2.63 (1.95–3.53)
35–39.9	26 (9.3)	3.11 (2.04–4.75)		2.44 (1.61–3.70)	2.36 (1.56–3.58)
≥ 40	16 (14.3)	4.63 (2.82–7.60)		3.21 (1.95–5.29)	3.15 (1.90–5.24)
Chronic hypertension					
No	319 (5.4)	1.00		1.00	1.00
Yes	17 (19.8)	3.64 (2.35–5.65)		1.74 (1.13–2.69)	1.70 (1.08–2.67)
Chronic diabetes					
No	319 (5.4)	1.00		1.00	1.00
Yes	17 (21.0)	3.86 (2.49–5.97)		2.31 (1.48–3.60)	2.29 (1.42–3.69)
Asthma requiring regular corticosteroids					
No	319 (5.5)	1.00		1.00	1.00
Yes	17 (9.3)	1.69 (1.06–2.70)		1.48 (0.92–2.38)	1.43 (0.90–2.29)
Multiple pregnancy					
No	327 (5.6)	1.00		1.00	1.00
Yes	10 (9.4)	1.70 (0.93–3.09)		1.41 (0.77–2.59)	1.35 (0.73–2.48)
Smoker during pregnancy					
No	294 (5.7)	1.00		1.00	1.00
Yes	16 (3.6)	0.63 (0.38–1.03)		0.79 (0.47–1.32)	0.88 (0.53–1.46)
Infection characteristics					
Period of infection					
March to May 2020	127 (8.6)	2.10 (1.22–3.59)			2.04 (1.20–3.47)
June to August 2020	14 (4.1)	1.00			1.00
September to November 2020	110 (3.4)	0.82 (0.48–1.42)			0.92 (0.54–1.58)
March to April 2021	86 (9.3)	2.26 (1.30–3.92)			1.96 (1.13–3.39)
Trimester of pregnancy					
T1 (0–14^+6^ WG)	11 (1.4)	1.00			1.00
Early T2 (15^+0^–21^+6^ WG)	15 (2.0)	1.44 (0.67–3.11)			1.40 (0.66–2.98)
Late T2 (22^+0^–28^+6^ WG)	103 (9.4)	6.64 (3.59–12.29)			5.71 (3.11–10.46)
T3 (29^+0^ WG to 6 days before delivery)	106 (4.9)	3.46 (1.87–6.40)			3.30 (1.80–6.04)
5 days before delivery to 7 days postpartum	101 (8.2)	5.79 (3.13–10.71)			4.81 (2.59–8.93)

*Note:* Crude and adjusted RR (95% CI) values were estimated using modified Poisson regression models after multiple imputation of missing data by chained equations.

Abbreviations: aRR, adjusted risk ratio; BMI, body mass index; CI, confidence interval; RR, risk ratio; T1, first trimester of pregnancy; T2, second trimester of pregnancy; T3, third trimester of pregnancy; WG, weeks of gestation.

^a^ % of severe infection in each subgroup of women.

### 3.4 Delivery, Maternal and Neonatal Outcomes According to Infection Severity

Half of women with severe COVID‐19 had a caesarean delivery, more than two‐thirds of caesareans being performed before labour in an emergency context. By contrast, the caesarean rates were less than 25% in all other COROPREG groups and in the ENP reference population (Table 4). General anaesthesia was required for nearly one fifth of women with severe COVID‐19 and in less than 2% in all other COROPREG groups as in the ENP reference population (Table 4). Three maternal deaths were due to severe COVID‐19 with acute respiratory distress (0.05% of all COROPREG population, 8.9% of women with severe COVID‐19). Another maternal death by haemorrhagic stroke occurred 7 days post‐partum in a woman with positive PCR screening at delivery and asymptomatic SARS‐CoV‐2 infection and was considered not related to SARS‐CoV‐2 infection by the team in charge.

**TABLE 4:** Delivery, maternal and neonatal outcomes by severity of SARS‐CoV‐2 infection.

	*N* ^a^	Severe infection	Non‐severe symptomatic infection	Non‐severe infection, symptoms not known	Asymptomatic infection	Overall COROPREG population	Reference population^e^
		*N* = 337	*N* = 3871	*N* = 681	*N* = 1126	*N* = 6015	*N* = 8057
Miscarriage. No. (%)^b^	5986	1 (0.3)	25 (0.6)	11 (1.6)	14 (1.2)	51 (0.9)	NA
Medical termination. No. (%)^b^	5986	0 (0.0)	11 (0.3)	3 (0.4)	2 (0.2)	16 (0.3)	NA
Among women who delivered	5920	333	3821	*658*	1108	5920	
Induction of labour. No. (%)	5894	94 (28.2)	1051 (27.6)	148 (22.7)	274 (24.8)	1567 (26.6)	26.1
Mode of delivery. No. (%)	5894						
Vaginal		168 (50.5)	2893 (76.1)	518 (79.3)	827 (74.8)	4406 (74.8)	78.9
Caesarean during labour		38 (11.4)	426 (11.2)	63 (9.7)	121 (10.9)	648 (11.0)	10.4
Caesarean before labour		127 (38.1)	483 (12.7)	72 (11.0)	158 (14.3)	840 (14.2)	10.7
Planned		16 (4.8)	273 (7.2)	40 (6.1)	103 (9.3)	432 (7.3)	6.9
Emergency		110 (33.0)	208 (5.5)	31 (4.7)	54 (4.9)	403 (6.8)	3.0
Not known		1 (0.3)	2 (0.1)	1 (0.2)	1 (0.1)	5 (0.1)	0.8
General anaesthesia. No. (%)	5840	59 (17.8)	50 (1.3)	9 (1.4)	12 (1.1)	130 (2.2)	1.2
Preeclampsia. No. (%)	5857	15 (4.5)	82 (2.2)	15 (2.3)	19 (1.7)	131 (2.2)	2.2
Postpartum haemorrhage. No. (%)^c^	5868	29 (8.9)	193 (5.1)	37 (5.7)	39 (3.5)	298 (5.1)	11.5
Postpartum haemorrhage> 1500 mL		4 (1.2)	14 (0.4)	5 (0.8)	4 (0.4)	27 (0.5)	0.6
Thrombotic event. No. (%)	5845	1 (0.3)	14 (0.4)	2 (0.3)	1 (0.1)	18 (0.3)	NA
Postpartum hospitalisation (days) median (IQR)	5841	5 (3–9)	3 (3–4)	3 (3–4)	3 (3–4)	3 (3–4)	3 (3–4)
Among newborns	6027	343	3897	666	1121	6027	
Gestational age at birth (WG). No. (%)	6015						
< 28^+0^		18 (5.2)	16 (0.4)	0 (0.0)	9 (0.8)	43 (0.7)	0.7
28 + 0–31^+6^		28 (8.2)	38 (1.0)	8 (1.2)	11 (1.0)	85 (1.4)	0.8
32 + 0–36^+6^		85 (24.8)	228 (5.9)	38 (5.7)	57 (5.1)	408 (6.8)	5.7
37 + 0–38^+6^		92 (26.8)	932 (24.0)	131 (19.7)	268 (24.0)	1423 (23.7)	23.2
≥ 39^+0^		120 (35.0)	2675 (68.8)	488 (73.4)	773 (69.1)	4056 (67.4)	69.5
Induced birth^d^ among births < 37 WG. No. (%)	533	113 (86.3)	170 (60.9)	27 (58.7)	43 (55.8)	353 (66.2)	49.1
Stillbirth. No. (%)	6000	12 (3.5)	21 (0.5)	1 (0.2)	10 (0.9)	44 (0.7)	0.6
Among livebirths	5957	331	3859	659	1108	5957	
Sex (%girls) No. (%)	5923	157 (47.4)	1904 (49.6)	305 (47.0)	543 (49.2)	2909 (49.1)	48.0
Birth weight (g). Mean (SD)	5936	2893 (804)	3266 (546)	3325 (551)	3271 (554)	3252 (572)	3255 (553)
SARS‐CoV2 PCR performed. No. (%)	5874	133 (41.0)	430 (11.3)	46 (7.2)	165 (15.1)	774 (13.2)	NA
Positive		13 (9.9)	33 (8.3)	1 (2.4)	12 (7.6)	59 (8.1)	NA
Hospitalisation. No. (%)	5941	156 (47.3)	444 (11.5)	78 (11.9)	126 (11.4)	804 (13.5)	10.7
For Sars‐CoV‐2 infection		4 (1.2)	3 (0.1)	0 (0.0)	2 (0.2)	9 (0.2)	
Hospitalisation in NICU. No. (%)	5941	73 (22.1)	120 (3.1)	28 (4.3)	24 (2.2)	245 (4.1)	2.9
For Sars‐CoV‐2 infection		2 (0.6)	1 (0.0)	0 (0.0)	0 (0.0)	3 (0.1)	
Neonatal death. No. (%)	5919	3 (0.9)	5 (0.1)	1 (0.2)	2 (0.2)	11 (0.2)	NA
Initial breastfeeding (exclusive or mixed). No. (%)	5794	191 (65.6)	2975 (78.7)	529 (82.7)	869 (80.2)	4564 (78.8)	71.2

Abbreviations: ENP, *Enquête Nationale Périnatale*; IQR, interquartile range; NICU, neonatal intensive care unit; WG, weeks of gestation.

^a^ Total number of available observations for each variable.

^b^ None related to SARS‐CoV‐2 infection.

^c^ Postpartum blood loss > 500 mL.

^d^ Induction of labour or caesarean before labour.

^e^ Women without SARS‐CoV‐2 infection from the ENP 2021 population restricted to COROPREG regions and with weights assigned in order to accurately represent the source population of the COROPREG survey.

Stillbirth occurred in 3.5% of women with severe COVID‐19 and in less than 1% in all other COROPREG groups and in the ENP reference population (Table 4). Among the 44 stillbirths, 6 were considered related to SARS‐CoV‐2 infection as assessed by the team in charge based on all available information and analyses, including 4 in a context of severe maternal symptoms and 2 non‐severe maternal symptoms (Table S3). There were about five times more preterm (< 37 WG) and ten times more very preterm births (< 32 WG) among women with severe COVID‐19 than in all other COROPREG groups and in the ENP reference population. In women with severe COVID‐19, more than 80% of preterm births were induced. A SARS‐CoV‐2 test was performed in 13.2% (*N* = 774) of neonates in the COROPREG population; 8.1% (*n* = 59) were positive and 3 infants were admitted to a neonatal intensive care unit. Finally, 2/3 of infants whose mothers had severe COVID‐19 were exclusively or partially breastfed, whereas 4/5 of infants whose mothers had non‐severe infection and those from the ENP reference population (Table 4).

## 4 Comment

### 4.1 Principal Findings

In our population‐based study of women with SARS‐CoV‐2 infection during pregnancy that used an organ dysfunction‐based definition of severity, severe COVID‐19 affected 1 to almost 4 women per 1000 deliveries and 4%–9% of infected women over the successive pandemic waves. Among women with SARS‐CoV‐2 infection, severe COVID‐19 was more frequent in those with social and medical vulnerability factors and in those infected in the second half of pregnancy. Neonatal adverse outcomes seemed to be confined to severe maternal infections and at least to some extent, attributable to induced prematurity.

### 4.2 Strengths of the Study

Strengths of the study include the population‐based design covering 60% of the French deliveries, including the regions at the frontline of the pandemic, with inclusion of all cases of SARS‐CoV‐2 infection during pregnancy, hospitalised or not, symptomatic or not, which is rare [14]. The population‐based estimations of severe infection rates using two denominators i.e., deliveries and cases of infection allowed us to minimise selection bias that may have resulted from missing out totally asymptomatic women who were not tested and whose infection remained unknown. Because the study was not limited to infected women admitted to the hospital or to intensive care, we were able to describe the profile of infected women and disease in a sample not biased by practice‐based selection criteria. In addition, the study was not limited to women with a positive PCR test and also involved those with a probable infection, ensuring that all cases were identified especially at the beginning of the pandemic when universal testing was not available. Our study collected detailed clinical and biological data for pregnant women with SARS‐CoV‐2 infection, often not available in other population‐based studies derived from administrative datasets [15]. Another strength was our choice of an organ dysfunction‐based definition of severe COVID‐19 rather than a management‐based definition, which tends to vary among places and over time and imperfectly reflects the severity of the disease. Finally, we were able to compare the COROPREG population to a reference population of women without infection in the same regions.

### 4.3 Limitations of the Data

The study was complex to set up and we regret the lack of a preexisting permanent surveillance system like those existing in other countries that would have been helpful to rapidly assess severe, maternal and neonatal morbidity [16, 17, 18]. Unfortunately, the complex coordination of this ad hoc study involving six regions and 281 units and the lack of further funding in 2020 prevented us from continuing to include women in all regions initially involved. However, the profile of infection rates was similar when limiting the analysis to women from regions with available data for the four study periods. We also could not include women in subsequent waves or assess the effect of vaccination and subsequent variants of concern [19, 20, 21]. Finally, test availability and testing policies evolved during the pandemic waves, which may have affected the inclusion of asymptomatic women in our study, but including women with a probable infection minimised this bias [22].

### 4.4 Interpretation

#### 4.4.1 Rate of Severe Forms

SARS‐CoV‐2 infection during pregnancy is a high‐risk situation; severe COVID‐19 occurred in 6% of infected pregnant women overall in our study and up to 9% in the first and third infection waves. Previous population‐based reports including only women admitted to hospital reported higher rates of severe forms, from 9% to 14% [16, 21, 23], probably overestimating the severity in the total population of infected women because they likely overlooked the least symptomatic or asymptomatic cases. Other large studies not limited to hospitalised women reported rates of severe forms similar to ours, but because they were not population‐based and included cases declared by centers, the external validity of their results is more questionable [24]. In addition, most previous studies used definitions of severity mainly or exclusively based on management criteria that vary across time and settings, which again highlights the importance of using an organ‐based definition of severity. One such management‐based severity criterion, often used, is intensive care unit admission, whose rates were generally higher (6%–18%) [15, 16] than the rate we report (about 3%), but, again, most studies included only hospital admissions, thus overestimating the severity. Finally, our 6% proportion of severe COVID‐19 is higher than the 2% proportion reported in the general all‐age population [25], which suggests a particular severity of SARS‐CoV‐2 infection in pregnant women, similar to what was previously reported for influenza [26].

#### 4.4.2 Risk Factors for Severe COVID‐19

Risk factors of severe COVID‐19 were mainly those of medical and social vulnerability that were already identified in the general population but also during the H1N1 influenza epidemic in pregnancy [27, 28, 29]: women from ethnic minorities, with impaired access to care, those who were younger or older, who were obese and had preexisting conditions should be carefully monitored in the event of future pandemics. In addition, infection ≥ 22 WG as compared with the first trimester was associated with severe COVID‐19, although previous, non‐population‐based studies reported severe COVID‐19 occurring in the third trimester only [30]. This increased risk could be explained by physiological changes including cardiac and respiratory changes with increased oxygen consumption and increased cardiac output in pregnancy [31] as well as decreased pulmonary volume leading to a physiological restrictive syndrome, all evolving during pregnancy. Changes in the immune system that help women tolerate the fetus may also affect the severity of the disease [32]. We also found an increased risk of severe illness with an increased number of people living in the household. So not only people living in such households were at increased risk of SARS‐CoV‐2 infection as previously reported but also at increased risk of severe COVID‐19. This situation may reflect a higher viral load in particular in the presence of young children as well as a delay in reaching care after initial symptoms [33].

#### 4.4.3 Obstetric Interventions and Neonatal Morbidity

In our study, the increased incidence of neonatal morbidity seemed confined to severe maternal disease. This morbidity mainly consisted of prematurity that was largely induced by management in that the maternal–fetal transmission of SARS‐CoV‐2 is known to be limited and rarely detrimental [34, 35]. Indeed, the high rate of urgent preterm caesarean section performed under general anaesthesia was similar to that in women presenting severe maternal morbidity of any cause before delivery [36]. These findings raise the question of obstetric interventions in women with severe COVID‐19 and in particular the indication for caesarean section. The general belief that pregnancy may compromise appropriate resuscitation care for women with severe illness, whatever the cause, may lead to decisions for emergency preterm caesarean section to facilitate optimal resuscitation such as prone position for oxygenation. However, the evidence that urgent preterm caesarean section reduces overall maternal and perinatal morbidity and mortality in case of severe illness is weak [37]. Extracorporeal membrane oxygenation was found feasible early in pregnancy, and its use may be considered for women at the lowest gestational ages [38]. Further analyses of data collected during the COVID‐19 pandemic on the association between maternal management and outcomes for both the woman and the infant, eventually combining data from different studies, will help inform appropriate practices for future pandemics [39].

## 5 Conclusions

Although an ad hoc population‐based cohort study provided unbiased results, which are important for assessing the impact of the COVID‐19 pandemic on vulnerable populations, the complexity of setting up this type of study remains an obstacle. Permanent perinatal surveillance systems need to be established to rapidly assess the impact of future pandemics and guide management, particularly in pregnant women, for whom severe forms of COVID‐19 are not uncommon.

## Author Contributions

CDT and PYA conceptualized the research, obtained funding, and coordinated the study. CDT, PYA, AS, CDu, CG, ML, EL, MPB and CVF designed the study protocol. NB,CC, CCH, CDu, CG, CL, ML, EM, VR coordinated the implementation of the study and collection of data in their region. AS and CDi conducted the statistical analysis. All authors contributed to the interpretation of the findings. CDT, AS, PYA and CDi led the writing of the original draft manuscript. All authors reviewed and edited the manuscript and approved the final version.

## Disclosure

*Permission to Reproduce Material From Other Sources*: NA.

## Consent

Oral consent was required to collect data from medical files. No written consent was necessary because of no intervention, in agreement with French law.

## Conflicts of Interest

The authors declare no conflicts of interest.

## Supporting information


**Figure S1.** Flow chart of the population study.

**Table S1.** Rates of SARS‐CoV‐2 infection, hospitalisation, severe infection and maternal intensive care unit admission by study period.
**TABLE S2.** Number and proportion of missing data for all reported variables, overall and by severity of SARS‐CoV2 infection.
**TABLE S3.** Description of the 44 stillbirths in the COROPREG population (*N* = 5920 women who delivered).

## Data Availability

Data are available on request, after an evaluation of a project proposal (email pierre-yves.ancel@inserm.fr or catherine.deneux-tharaux@inserm.fr).
