# Supplementary material for: Incidence, Risk Factors and Outcomes of SARS‐CoV‐2 Infection in Pregnant Women: The COROPREG Population‐Based Study
Source: Paediatr Perinat Epidemiol. 2025 May 21;39(5):477–94. doi: 10.1111/ppe.70028 (PMC12308625; doi:10.1111/ppe.70028)
Supplement: Supplementary file 1 — Figure S1. Flow chart of the population study. [file PPE-39-477-s001.pptx]

## Slide 1
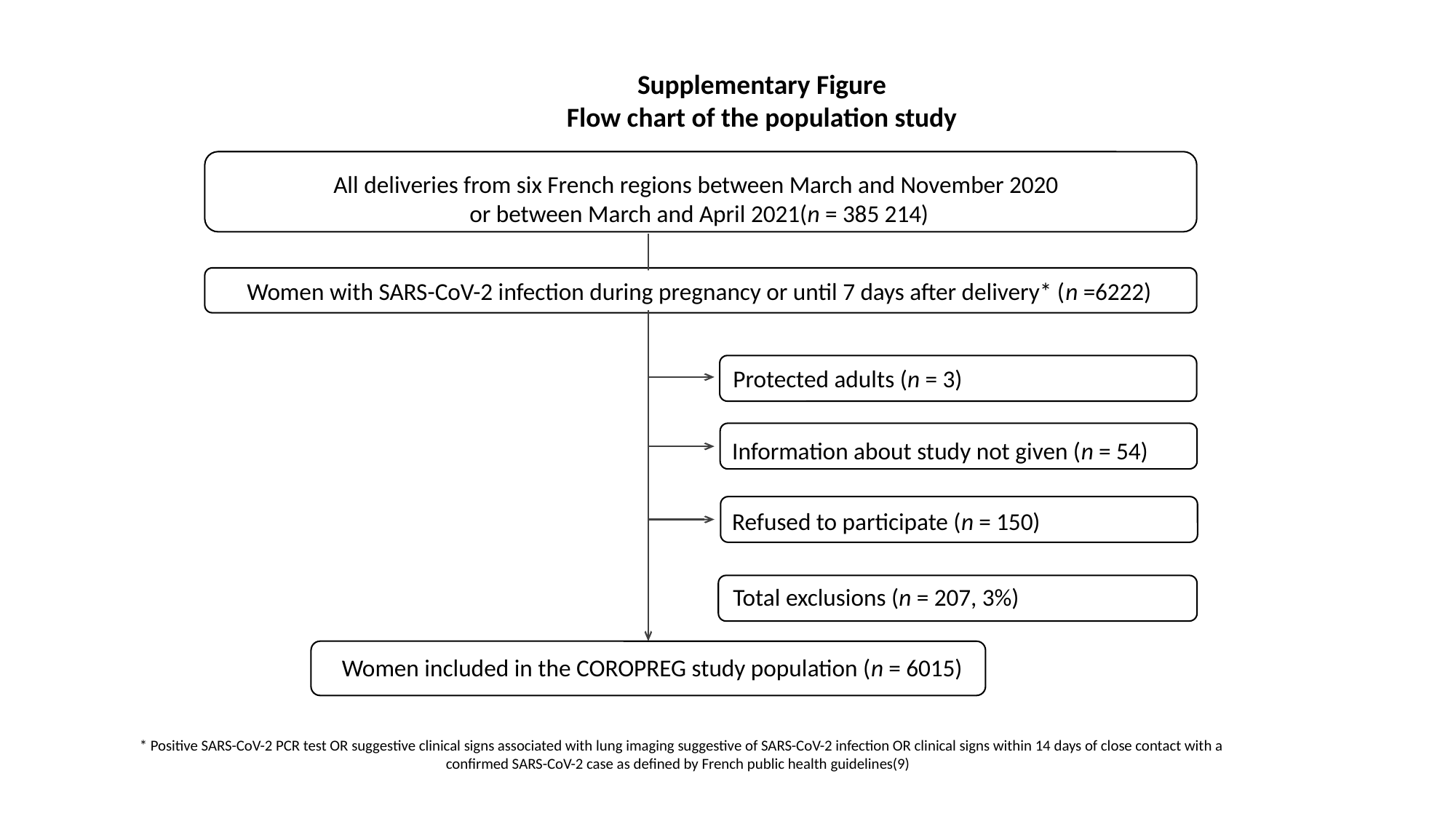

Supplementary Figure
Flow chart of the population study
All deliveries from six French regions between March and November 2020
or between March and April 2021(n = 385 214)
Protected adults (n = 3)
Information about study not given (n = 54)
Refused to participate (n = 150)
Total exclusions (n = 207, 3%)
Women included in the COROPREG study population (n = 6015)
Women with SARS-CoV-2 infection during pregnancy or until 7 days after delivery* (n =6222)
* Positive SARS-CoV-2 PCR test OR suggestive clinical signs associated with lung imaging suggestive of SARS-CoV-2 infection OR clinical signs within 14 days of close contact with a confirmed SARS-CoV-2 case as defined by French public health guidelines(9)
