# Supplementary material for: Incidence, Risk Factors and Outcomes of SARS‐CoV‐2 Infection in Pregnant Women: The COROPREG Population‐Based Study
Source: Paediatr Perinat Epidemiol. 2025 May 21;39(5):477–94. doi: 10.1111/ppe.70028 (PMC12308625; doi:10.1111/ppe.70028)
Supplement: Supplementary file 2 — Table S1. Rates of SARS‐CoV‐2 infection, hospitalisation, severe infection and maternal intensive care unit admission by study period. TABLE S2. Number and proportion of missing data for all reported variables, overall and by severity of SARS‐CoV2 infection. TABLE S3. Description of the 44 stillbirths in the COROPREG population (N = 5920 women who delivered). [file PPE-39-477-s002.docx]

Supplementary Table 1: Rates of SARS-CoV-2 infection, hospitalization, severe infection and maternal intensive care unit admission by study period

|  | **March-May 2020** | **June-August 2020** | **September-November 2020** | **March-April 2021** |
| --- | --- | --- | --- | --- |
| **Number of deliveries** | 117386 | 123527 | 120587 | 23714 |
| **SARS-CoV-2 infection** |  |  |  |  |
| Number | 1480 | 342 | 3264 | 929 |
| Rate per 1000 deliveries (95% CI) | 12.6 (11.9-13.3) | 2.8 (2.5-3.1) | 27.1 (26.2-28.0) | 39.2 (36.7-41.7) |
| **Hospitalization with SARS-CoV-2 infection** |  |  |  |  |
| Number | 380 | 57 | 334 | 195 |
| Rate per 1000 deliveries (95% CI) | 3.2 (2.9-3.6) | 0.5 (0.4-0.6) | 2.8 (2.5-3.1) | 8.2 (7.1-9.5) |
| Rate per 100 SARS-CoV-2 cases (95% CI) | 25.7 (23.5-28.0) | 16.7 (12.9-21.1) | 10.2 (9.2-11.3) | 21.1 (18.4-23.7) |
| **Severe SARS-CoV-2 infection^a^** |  |  |  |  |
| Number | 127 | 14 | 110 | 86 |
| Rate per 1000 deliveries (95% CI) | 1.1 (0.9-1.3) | 0.1 (0.1-0.2) | 0.9 (0.8-1.1) | 3.6 (2.9-4.5) |
| Rate per 100 SARS-CoV-2 cases (95% CI) | 8.6 (7.2-10.1) | 4.1 (2.3-6.8) | 3.4 (2.8-4.0) | 9.3 (7.5-11.3) |
| **Hospitalization in intensive care with SARS-CoV-2** |  |  |  |  |
| Number | 74 | 11 | 70 | 48 |
| Rate per 1000 deliveries (95% CI) | 0.6 (0.5-0.8) | 0.1 (0.0-0.2) | 0.6 (0.5-0.7) | 2.0 (1.5-2.7) |
| Rate per 100 SARS-CoV-2 cases (95% CI) | 5.0 (4.0-6.2) | 3.2 (1.6-5.7) | 2.1 (1.7-2.7) | 5.2 (3.8-6.8) |

CI: confidence interval

^a^ Severe infection was defined by at least one of arterial pH <7.38, lactate level >2.0 mmol/L, PaO2 ≤ 80 mmHg, PaO2/FIO2 <300, continuous positive airway pressure, high-flow oxygen therapy, invasive mechanical ventilation, clinical diagnosis of acute respiratory distress requiring oxygen therapy, prone position, extracorporeal membrane oxygenation, platelet count <70000/mm3, prothrombin time <60%, plasmatic fibrinogen level <2 g/l, creatinine level ≥110 μmol/L, dialysis, catecholamines administration

Supplementary Table 2: Number and proportion of missing data for all reported variables, overall and by severity of SARS-CoV2 infection

|  | **Overall COROPREG population** | **Severe infection** | **Non-severe symptomatic infection** | **Non-severe infection, symptoms not known** | **Asymptomatic** |
| --- | --- | --- | --- | --- | --- |
|  | **N=6015** | **N=337** | **N=3871** | **N=681** | **N=1126** |
|  | **n (%)** | **n (%)** | **n (%)** | **n (%)** | **n (%)** |
| **SARS-CoV-2 infection** |  |  |  |  |  |
| Time of SARS-CoV-2 infection diagnosis | 15 (0.3) | 2 (0.6) | 9 (0.2) | 2 (0.2) | 2 (0.3) |
| SARS-CoV-2 diagnosis | 0 (0.0) | 0 (0.0) | 0 (0.0) | 0 (0.0) | 0 (0.0) |
| Severity of infection | 0 (0.0) | 0 (0.0) | 0 (0.0) | 0 (0.0) | 0 (0.0) |
| Symptoms among symptomatic infections | 681 (11.3) | 0 (0.0) | 0 (0.0) | 681 (100) | 0 (0.0) |
| Cough | 93 (2.2) | 3 (0.9) | 90 (2.3) | - | 0 (0.0) |
| Fever or fever feeling | 86 (2.0) | 2 (0.6) | 84 (2.2) | - | 0 (0.0) |
| Loss or decrease of smell or taste | 100 (2.4) | 14 (4.3) | 86 (2.2) | - | 0 (0.0) |
| Severe fatigue or lethargy | 116 (2.8) | 10 (3.1) | 106 (2.7) | - | 0 (0.0) |
| Headache | 148 (3.5) | 15 (4.6) | 133 (3.4) | - | 0 (0.0) |
| Rhinorrhea | 142 (3.4) | 16 (4.9) | 126 (3.3) | - | 0 (0.0) |
| Joint or muscular pain | 135 (3.2) | 15 (4.6) | 120 (3.1) | - | 0 (0.0) |
| Dyspnea | 97 (2.3) | 1 (0.3) | 96 (2.5) | - | 0 (0.0) |
| Sore throat | 161 (3.8) | 18 (5.5) | 143 (3.7) | - | 0 (0.0) |
| Nausea or vomiting | 121 (2.9) | 8 (2.5) | 113 (2.9) | - | 0 (0.0) |
| Diarrhea | 124 (3.0) | 10 (3.1) | 114 (2.9) | - | 0 (0.0) |
| Chest pain | 124 (3.0) | 11 (3.4) | 113 (2.9) | - | 0 (0.0) |
| Other | 84 (2.0) | 5 (1.5) | 79 (2.0) | - | 0 (0.0) |
| Maternal hospitalization with SARS-CoV-2 infection | 28 (0.5) | 1 (0.3) | 12 (0.3) | 13 (1.9) | 2 (0.2) |
| Maternal ICU hospitalization with SARS-CoV-2 infection | 26 (0.4) | 1 (0.3) | 11 (0.3) | 12 (1.8) | 2 (0.2) |
| Maternal death | 0 (0.0) | 0 (0.0) | 0 (0.0) | 0 (0.0) | 0 (0.0) |
| **Socio-demographic characteristics** |  |  |  |  |  |
| Age | 3 (0.0) | 0 (0.0) | 0 (0.0) | 2 (0.3) | 1 (0.1) |
| Region or country of birth | 310 (5.2) | 16 (4.7) | 169 (4.4) | 51 (7.5) | 74 (6.6) |
| Profession in contact with the public | 807 (13.4) | 66 (19.6) | 430 (11.1) | 126 (18.5) | 185 (16.4) |
| No standard healthcare insurance | 460 (7.6) | 34 (10.1) | 280 (7.2) | 77 (11.3) | 69 (6.1) |
| Living without a partner | 599 (10.0) | 33 (9.8) | 359 (9.3) | 113 (16.6) | 94 (8.4) |
| Unemployment at the beginning of pregnancy | 708 (11.8) | 48 (14.2) | 380 (9.8) | 116 (17.0) | 164 (14.6) |
| No personal housing | 846 (14.1) | 47 (13.9) | 534 (13.8) | 146 (21.4) | 119 (10.6) |
| More than 4 people living in the housing | 1203 (20.0) | 71 (21.1) | 760 (19.6) | 167 (24.5) | 205 (18.2) |
| Smoking during pregnancy | 397 (6.6) | 27 (8.0) | 205 (5.3) | 87 (12.8) | 78 (6.9) |
| **Medical History** |  |  |  |  |  |
| BMI | 268 (4.5) | 25 (7.4) | 142 (3.7) | 57 (8.4) | 44 (3.9) |
| Chronic hypertension | 61 (1.0) | 1 (0.3) | 25 (0.6) | 29 (4.3) | 6 (0.5) |
| Diabetes | 61 (1.0) | 1 (0.3) | 25 (0.6) | 29 (4.3) | 6 (0.5) |
| Asthma requiring regular inhaled or oral corticosteroids | 64 (1.1) | 1 (0.3) | 25 (0.6) | 30 (4.4) | 8 (0.7) |
| Chronic heart disease | 64 (1.1) | 1 (0.3) | 27 (0.7) | 29 (4.3) | 7 (0.6) |
| Autoimmune disease | 64 (1.1) | 1 (0.3) | 27 (0.7) | 29 (4.3) | 7 (0.6) |
| Chronic inflammatory disease | 64 (1.1) | 1 (0.3) | 27 (0.7) | 29 (4.3) | 7 (0.6) |
| Acquired or induced immunodepression | 60 (1.0) | 1 (0.3) | 24 (0.6) | 29 (4.3) | 6 (0.5) |
| **Pregnancy characteristics** |  |  |  |  |  |
| Parity | 44 (0.7) | 1 (0.3) | 12 (0.3) | 27 (4.0) | 4 (0.4) |
| Multiple pregnancy | 14 (0.2) | 0 (0.0) | 2 (0.1) | 9 (1.3) | 3 (0.3) |
| In vitro fertilization | 175 (2.9) | 12 (3.6) | 95 (2.5) | 27 (4.0) | 41 (3.6) |
| **Delivery, maternal and neonatal** **characteristics** |  |  |  |  |  |
| Miscarriage | 29 (0.5) | 3 (0.9) | 15 (0.4) | 9 (1.3) | 2 (0.2) |
| Medical termination | 29 (0.5) | 3 (0.9) | 15 (0.4) | 9 (1.3) | 2 (0.2) |
| **Missing on any variable included in the multivariable analysis**^a^ | 1872 (31.1) | 129 (38.3) | 1171 (30.3) | 260 (38.2) | 312 (27.7) |
| **Women who delivered** | 29 (0.5) | 3 (0.9) | 15 (0.4) | 9 (1.3) | 2 (0.2) |
| Induction of labor | 26 (0.4) | 0 (0.0) | 18 (0.5) | 5 (0.8) | 3 (0.3) |
| Mode of delivery | 26 (0.4) | 0 (0.0) | 19 (0.5) | 5 (0.8) | 2 (0.2) |
| General anesthesia | 80 (1.4) | 2 (0.6) | 48 (1.3) | 19 (2.9) | 11 (1.0) |
| Preeclampsia | 63 (1.1) | 3 (0.9) | 40 (1.1) | 16 (2.4) | 4 (0.4) |
| Postpartum hemorrhage | 52 (0.9) | 6 (1.8) | 32 (0.8) | 13 (2.0) | 1 (0.1) |
| Thrombotic event | 75 (1.3) | 6 (1.8) | 48 (1.3) | 17 (2.6) | 4 (0.4) |
| Length of postpartum hospitalization | 79 (1.3) | 14 (4.2) | 45 (1.2) | 16 (2.4) | 4 (0.4) |
| **Newborns** | 29 (0.5) | 3 (0.9) | 15 (0.4) | 9 (1.3) | 2 (0.2) |
| Gestational age at birth | 12 (0.2) | 0 (0.0) | 8 (0.2) | 1 (0.2) | 3 (0.3) |
| Induced birth among births <37 GW | 33 (0.5) | 0 (0.0) | 24 (0.6) | 6 (0.9) | 3 (0.3) |
| Stillbirth | 26 (0.4) | 0 (0.0) | 17 (0.4) | 6 (0.9) | 3 (0.3) |
| **Among livebirths** | 26 (0.4) | 0 (0.0) | 17 (0.4) | 6 (0.9) | 3 (0.3) |
| Sex | 34 (0.6) | 0 (0.0) | 19 (0.5) | 10 (1.5) | 5 (0.5) |
| Birth weight | 21 (0.4) | 1 (0.3) | 12 (0.3) | 3 (0.5) | 5 (0.5) |
| SARS-CoV2 PCR performed | 83 (1.4) | 7 (2.1) | 42 (1.1) | 18 (2.7) | 16 (1.4) |
| Positive SARS-CoV2 PCR | 126 (2.1) | 9 (2.7) | 72 (1.9) | 22 (3.3) | 23 (2.1) |
| Hospitalization | 16 (0.3) | 1 (0.3) | 10 (0.3) | 4 (0.6) | 1 (0.1) |
| Hospitalization in NICU | 16 (0.3) | 1 (0.3) | 10 (0.3) | 4 (0.6) | 1 (0.1) |
| Neonatal death | 38 (0.6) | 5 (1.5) | 25 (0.7) | 6 (0.9) | 2 (0.2) |
| Initial breastfeeding | 163 (2.7) | 37 (11.2) | 77 (2.0) | 21 (3.2) | 28 (2.5) |

ICU: intensive care unit, BMI: body mass index, NICU: neonatal intensive care unit

^a^ Maternal age, region or country of birth, living without a partner, standard healthcare insurance, personal housing, number of people living in the household, BMI, chronic hypertension, chronic diabetes, asthma requiring regular corticosteroids, multiple pregnancy, period of infection, trimester of pregnancy

Supplementary Table 3: Description of the 44 stillbirths in the COROPREG population (N=5920 women who delivered)

|  | **All stillbirths**  **N=44** | | **Stillbirth related to SARS-CoV-2 infection**  **N=6** |
| --- | --- | --- | --- |
|  | **N** | **%** | **N** |
| Time of diagnosis |  |  |  |
| Before labor | 40 | 90.9 | 6 |
| During labor | 4 | 9.1 | 0 |
| Cause^a^ |  |  |  |
| SARS-CoV-2 infection | 6 | 15.4 | 6 |
| Other than SARS-CoV-2 infection | 14 | 35.9 | 0 |
| None identified | 19 | 48.7 | 0 |
| **Maternal characteristics** |  |  |  |
| Timing of infection (trimester of pregnancy) |  |  |  |
| T1 (0-14+6 WG) | 2 | 4.6 | 0 |
| Early T2 (15+0-21+6 WG) | 8 | 18.2 | 0 |
| Late T2 (22+0-28+6 WG) | 20 | 45.5 | 3 |
| T3 (29+0 WG to 6 days before delivery) | 4 | 9.1 | 2 |
| 5 days before delivery to 7 days postpartum | 10 | 22.7 | 1 |
| Period of infection |  |  |  |
| March-May 2020 | 9 | 20.4 | 0 |
| June-August 2020 | 0 | 0.0 | 0 |
| September-November 2020 | 23 | 52.3 | 4 |
| March-April 2021 | 12 | 27.3 | 2 |
| Severity of SARS-CoV-2 infection |  |  |  |
| Severe infection | 12 | 27.3 | 4 |
| Non-severe symptomatic infection | 21 | 47.7 | 2 |
| Non-severe infection, symptoms not known | 1 | 2.3 | 0 |
| Asymptomatic infection | 10 | 22.7 | 0 |
| Multiple pregnancy | 5 | 11.4 | - |
| Gestational age at stillbirth diagnosis (WG) |  |  |  |
| 22-23 | 11 | 25.0 | 1 |
| 24-25 | 4 | 9.1 | 1 |
| 26-27 | 1 | 2.3 | 0 |
| 28-31 | 11 | 25.0 | 2 |
| 32-36 | 6 | 13.6 | 1 |
| 37-38 | 6 | 13.6 | 1 |
| >38 | 5 | 11.4 | 0 |

WG, weeks of gestation

^a^ as assessed by the team in charge based on all available information and analyses
